# Protein vicinal thiols as intrinsic probes of brain redox states in health, aging, and ischemia

**DOI:** 10.1007/s11011-024-01370-3

**Published:** 2024-06-07

**Authors:** Timothy D. Foley, Wen C. Huang, Emily A. Petsche, Emily R. Fleming, James C. Hornickle

**Affiliations:** Biochemistry Program, Department of Chemistry, University of Scranton, Scranton, PA 18510 USA

**Keywords:** Aging, Disulfide bonds, Ischemia, Peroxiredoxin, Protein thiols, Redox

## Abstract

The nature of brain redox metabolism in health, aging, and disease remains to be fully established. Reversible oxidations, to disulfide bonds, of closely spaced (vicinal) protein thiols underlie the catalytic maintenance of redox homeostasis by redoxin enzymes, including thioredoxin peroxidases (peroxiredoxins), and have been implicated in redox buffering and regulation. We propose that non-peroxidase proteins containing vicinal thiols that are responsive to physiological redox perturbations may serve as intrinsic probes of brain redox metabolism. Using redox phenylarsine oxide (PAO)-affinity chromatography, we report that PAO-binding vicinal thiols on creatine kinase B and alpha-enolase from healthy rat brains were preferentially oxidized compared to other selected proteins, including neuron-specific (gamma) enolase, under conditions designed to trap in vivo protein thiol redox states. Moreover, measures of the extents of oxidations of vicinal thiols on total protein, and on creatine kinase B and alpha-enolase, showed that vicinal thiol-linked redox states were stable over the lifespan of rats and revealed a transient reductive shift in these redox couples following decapitation-induced global ischemia. Finally, formation of disulfide-linked complexes between peroxiredoxin-2 and brain proteins was demonstrated on redox blots, supporting a link between protein vicinal thiol redox states and the peroxidase activities of peroxiredoxins. The implications of these findings with respect to underappreciated aspects of brain redox metabolism in health, aging, and ischemia are discussed.

**Supplementary Information:**

The online version contains supplementary material available at 10.1007/s11011-024-01370-3.

## Introduction

Perturbations of oxido-reductive (redox) metabolism, giving rise to assumed global “oxidative stresses”, have long been hypothesized to contribute to brain dysfunction in aging, ischemia, and neurodegenerative disorders (Foley 2019). The generally accepted state of oxidative stress under these conditions has been informed mainly by measures of indiscriminate and irreversible, free radical-mediated, oxidations of biomolecules, the significance of which for brain function remains uncertain (Foley 2019). These oversimplified perspectives disregard (i) the diversity of redox couples that can be reversibly oxidized, and that may be more directly linked to cellular metabolism, and (ii) cellular compensatory mechanisms that can maintain redox homeostasis in response to oxidative insults and even give rise to reductive stresses in certain redox couples (Foley et al. 2019; Xiao and Loscalzo 2020).

Reversible oxidations, to disulfide bonds, of protein cysteinyl thiols that are closely spaced (defined here as vicinal) by virtue of primary, tertiary, or quaternary structure, have emerged at the center of redox homeostasis. In particular, dithiol-disulfide cycling underpins the catalytic preservation of redox homeostasis by redoxin enzymes, including thioredoxin peroxidases (peroxiredoxins) (Holmgren et al. 2005), and have been connected, more generally, to redox buffering and regulation. Indeed, findings by us (Foley et al. 2014) and others (Beer et al. 2004; Hansen et al. 2009) argue that protein disulfides form more readily than do mixed disulfides with glutathione (i.e., S-glutathionylation) under physiological conditions, consistent with the notion that protein thiols may be as crucial for cellular redox buffering as is glutathione (Hansen et al. 2009).

Measurements of the vicinal thiol redox states of Prxs, by redox blotting, have been employed as internal probes of redox metabolism in cells (Cox et al. 2010). However, the extraordinarily high reactivities of the peroxidatic thiols of Prxs with hydrogen peroxide can present challenges to trapping the in vivo redox states of these enzymes (Peskin et al. 2007). Indeed, we have observed unusually high extents of oxidations of Prx-2 and Prx-1 from brain under experimental conditions considered sufficient to trap the in vivo redox states of thiols on non-peroxidase proteins and on glutathione (Foley et al. 2014, 2016). Thus, we propose that measures of redox states of vicinal thiols on proteins other than peroxidases may prove advantageous as reporters of redox perturbations occurring in tissues, in vivo. Furthermore, measurements of the redox states of conserved vicinal thiols on *different isoforms* of proteins may provide feedback on cell and organelle-specific alterations in metabolism depending on the expression patterns of the proteins employed as redox probes.

We have developed and exploited redox phenylarsine oxide (PAO)-affinity chromatography to capture proteins containing arsenical-binding vicinal thiols that have undergone oxidations to disulfide bonds (Foley et al. 2010, 2014, 2016, 2020). This work has identified diverse proteins, including relatively abundant enzymes, from rat brain containing PAO-binding vicinal thiols that can be oxidized, fractionally, to operationally-defined albeit non-structural disulfide bonds. The present study compared the extents of oxidations of PAO-binding thiols on several of these enzymes from 100,000 × g supernatants from the brains of young healthy rats to establish which are most responsive to physiological oxidants generated in the brain and may be particularly useful as sensors of brain redox metabolism. Among the enzymes selected, alpha- and gamma-enolase isoforms were of interest because they offer potential insights into cell type-specific redox metabolism. Critically, brains were ordinarily flash frozen immediately following euthanasia, by decapitation, and reduced protein thiols were trapped by alkylation with N-ethylmaleimide (NEM) during tissue homogenization to prevent or limit postmortem changes in protein thiol redox states. The results highlight novel aspects of protein thiol-linked redox metabolism in the brain in health, aging, and in the early moments following decapitation-induced global ischemia.

## Materials and methods

### Materials

Pierce™ Protease Inhibitor Mini Tablets, Coomassie Protein Assay, Imperial™ Protein Stain, TCEP, and DTT were from Thermo-Fisher (Waltham, MA). Mini-Protean® TGX™ precast protein gels, nitrocellulose blotting membranes, Affi-Gel 10, Bio-Gel P-6, Laemmli sample buffer, and Mini Bio-Spin columns were from Bio-Rad (Hercules, CA). WesternSure® Premium chemiluminescent blotting substrate was from Li-Cor Biosciences (Lincoln, NE). All primary antibodies (sc-48345, sc-136178, sc-21738, sc100812, sc-365062, and sc-515428) and the mouse IgG kappa binding protein [m-IgGκ BP; (sc-516102)] conjugated to horseradish peroxidase (HRP), were from Santa Cruz Biotechnology (Santa Cruz, CA). All other reagents were from Millipore-Sigma (Burlington, MA).

### Collection and storage of the rat brains under study

Brains used for the comparison of thiol oxidations in the enzymes of interest and for the investigation of interprotein disulfide crosslinks involving Prx-2 were obtained from male Sprague–Dawley (SD) rats that were 4–5 weeks and 6–7 weeks old, respectively. Following euthanasia by decapitation, severed heads were dropped immediately into liquid nitrogen and brains were removed from the frozen heads using a chisel. In addition to these immediately-frozen brains, brains that had been frozen following intentional delays to freezing of 3 min and 15 min were also obtained from the 4–5-week-old rats. All frozen brains were transferred to a -80 °C freezer for storage prior to homogenizations and fractionations.

Brains used for the aging study were from Fischer 344 (F344) male rats, of 4, 18, and 28 months of age, and were obtained from the National Institute on Aging (NIA), shipped to us overnight on dry ice. According to NIA protocols, the animals had been euthanized by CO_2_ asphyxiation followed by cervical dislocation and brains had been removed within 5 min of anoxia and stored at -80 °C.

### Preparation of alkylated protein fractions

For all experiments other than the trapping of interprotein disulfide bonds involving Prx-2, brains were weighed, partially thawed, and homogenized, at 5 mL/g of tissue, in Tris–EDTA buffer (TEB; 200 mM Tris, 10 mM EDTA, 1 mM benzamidine, pH 7.0) to which was added Triton X-100 (1% v/v), N-ethylmaleimide (NEM; 200 mM), and a Pierce™ Protease Inhibitor Mini Tablet. As previously described in detail (Foley et al. 2014, 2016), homogenates were alkylated for 1 h at room temperature (21–23 °C) and centrifuged for 65 min at 100,000 × g at 4 °C to yield supernatants containing a combination of soluble and Triton X-100-solubilized protein.

For study of the interprotein disulfides involving Prx-2, brains were homogenized in 20 mM acetic acid, pH 4.0, containing a pre-dissolved Pierce™ Protease Inhibitor Mini Tablet. Aliquots of the homogenates were diluted 1:1 with TEB containing 1% (v/v) Triton X-100, and 1 mM benzamidine and either none, 0.2 M NEM, or 0.4 M NEM. Following a 30 min incubation period at 21–23 °C, to permit alkylation of protein thiols in NEM-containing samples, homogenates were centrifuged at 100,000 × g for 65 min at 4 °C. The resulting supernatants were diluted with the Tris–EDTA buffer to 1 mg protein/mL, further diluted 1:1 with 2x Laemmli sample buffer containing either no reducing agent or 20 mM DTT, and heated for 5 min at 95 °C.

### Fractionation of soluble proteins by redox PAO-affinity chromatography

All procedures were conducted, with minor modifications, as described in detail previously (Foley et al. 2020). Briefly, unreacted NEM was removed from the 100,000 × g supernatants by centrifugal gel filtration. One-mL volumes of NEM-free supernatants, containing 3 mg total protein, were incubated with immobilized PAO for 90 min at 21–23 °C followed by fractionation to yield flow-through (FT), washes, and DTT-eluted proteins formerly containing operationally-defined disulfide bonds. Aliquots of pre-column (Pre) fractions, together with the FT, last wash (LW) and DTT-eluted fractions were diluted 1:1 with 80% (v/v) glycerol and stored at -20 °C until analyzed.

### Measurements of protein

Protein concentrations in the 100,000 × g supernatants and in the DTT-eluted fractions generated during immobilized PAO-affinity chromatography were quantified, using the Coomassie Protein Assay (Thermo-Fisher), from standard curves generated using bovine serum albumin.

### Analyses of protein from the PAO-affinity fractions

To confirm the presence of the proteins of interest in the DTT-eluted fractions, a representative sample from an immediately frozen brain, contained in an excised but unresolved gel band, was shipped to MS Bioworks LLC (Ann Arbor, MI) for alkylation of reduced thiols with iodoacetamide, in-gel tryptic digestion, and protein identification by LC–MS/MS as described in detail earlier (Foley et al. 2020). Proteins in the gel band were alkylated with iodoacetamide, subjected to in-gel tryptic digestion, and identified by LC–MS/MS using a Waters NanoAcquity HPLC system interfaced to a ThermoFisher Q Exactive. Data were searched using Mascot and filtered using a 1% protein and peptide FDR and requiring at least two unique peptides per protein. Protein cysteine residues labeled by NEM were considered to contain thiols that were reduced in the brain. Protein cysteine residues labeled with iodoacetamide, giving rise to carbamidomethyl groups, were assumed to contain thiols that had been reversibly oxidized [i.e., Cys(ox)] prior to the on-column reduction of disulfide bonds by TCEP during the PAO-affinity fractionation described above. Analysis of oxidized and reduced thiols was performed using Scaffold software.

Protein gel electrophoresis and western blotting were performed as described previously (Foley et al. 2020). Blots were developed using WesternSure® Premium chemiluminescent blotting substrate and the C-DiGit Blot Scanner (Li-Cor Biosciences) and analyzed using Image Studio™ software (Li-Cor Biosciences).

### Prx-2 redox blots

Samples prepared as described above in non-reducing and reducing Laemmli sample buffer, and containing 5 µg protein each, were resolved on 4–20% precast gels and transferred to 0.2 µm nitrocellulose blotting membranes. After blocking with 5% (m/v) nonfat dry milk, blots were incubated overnight at 4 °C with primary antibody to Prx-2 at a 1:200 dilution, washed 3 × for 5 min each with Tris-buffered saline containing 0.1% Tween-20, and incubated for 2 h at room temp with HRP-conjugated secondary antibody at a 1:1000 dilution. Blots were developed as described above.

### Statistical analyses

For study of the effects of delays to tissue freezing on protein thiol redox states, brains that had been immediately-frozen, and frozen following delays of 3 and 15 min, were experimentally paired by processing one brain from each of the three groups on days of tissue fractionations. Statistically-significant differences of the means (*N* = 5) between the control (immediately-frozen) and the delayed groups, and between the 3 and 15 min groups, were assessed by paired student t-test. Statistically-significant differences among the means (*N* = 3–5) for the three age groups for total protein, creatine kinase B, and alpha-enolase were assessed by one-way ANOVA.

## Results

### Creatine kinase B and alpha-enolase, but not gamma-enolase, contain thiols that are preferentially oxidized under conditions designed to trap in vivo thiol redox states

Proteins from 100,000 × g supernatants of the brains under study were fractionated by redox PAO-affinity chromatography. Like other trivalent arsenicals, PAO binds with high affinity to closely-spaced pairs of thiols giving rise to dithioarsine rings (Adams et al. 1990). The redox PAO-affinity method involves alkylation of reduced thiols, in this study by NEM, followed by on-column reduction of protein disulfides by TCEP, making available PAO-binding thiol pairs only on the proteins that contained reducible disulfide bonds. Following displacement by DTT, the bound proteins, formerly containing disulfide bonds, can be analyzed.

**Fig. 1:**
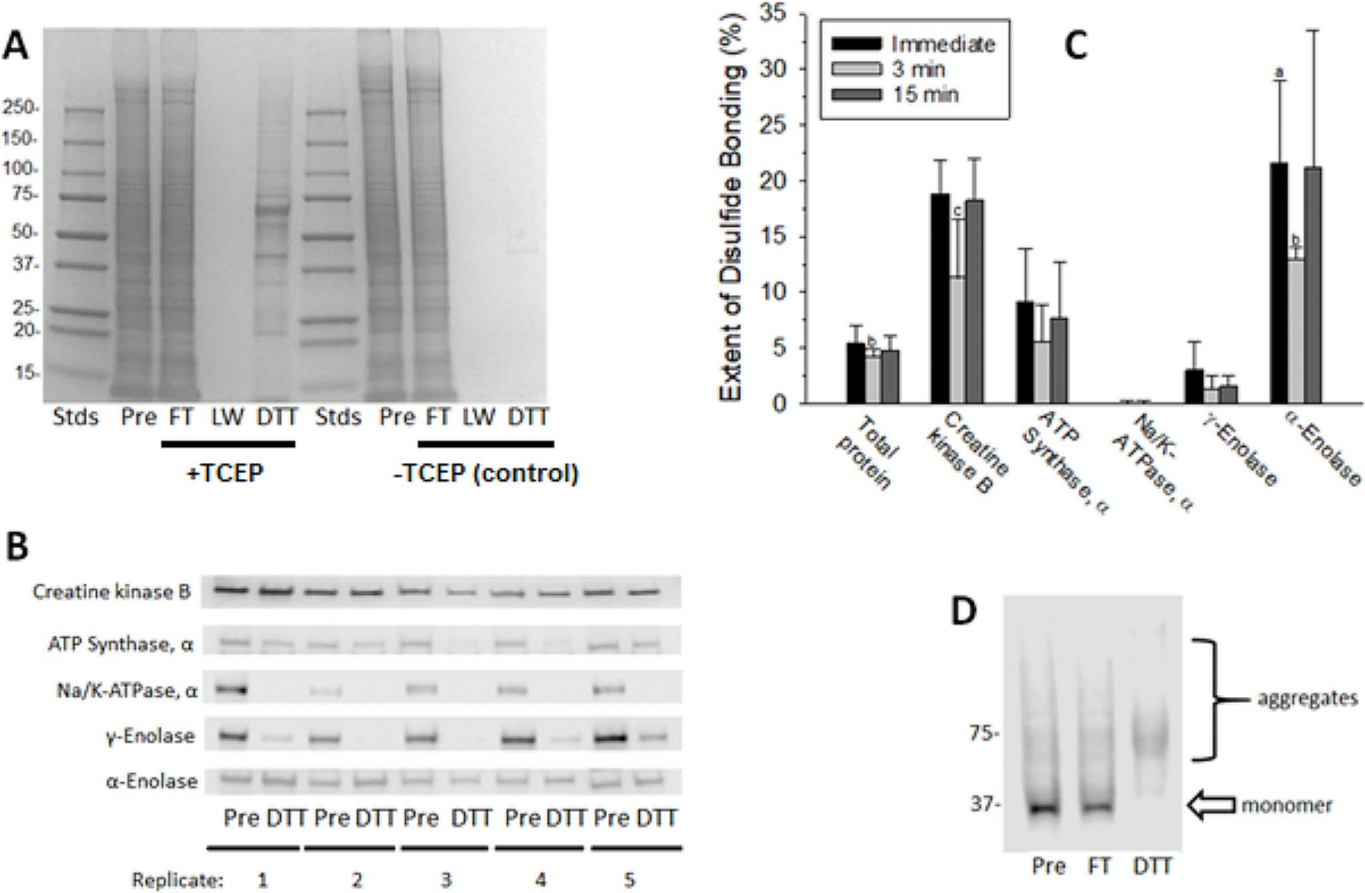
Creatine kinase B and alpha-enolase contain thiols that are preferentially oxidized under conditions designed to trap in vivo thiol redox states. Rat brains that had been flash-frozen immediately following decapitation, and after post-decapitation periods of 3 and 15 min, were removed from storage at -80 °C, homogenized in the presence of NEM and Triton X-100, and incubated for 1 h to allow alkylation of reduced protein thiols. Following centrifugation to yield 100,000 × g supernatants and removal of unreacted NEM, proteins were fractionated by redox PAO-affinity chromatography. **A** A gel stained for total protein with Imperial™ Protein Stain showing the results of a representative redox PAO-affinity fractionation of an immediately-frozen brain. The molecular weights of standards (Stds) are given in kDa. Pre (pre-column, total protein), flow-through (FT, unbound protein); last wash (LW), and DTT-eluted (disulfide bond-containing) fractions are labeled. **B** Western blots, imaged by chemiluminescence for the enzymes of interest, in the Pre and DTT fractions from fractionations of five immediately-frozen brains. For **A** and **B**, the DTT fractions were concentrated 5 × relative to the Pre fractions to aid visualization and measurement. **C** The percentages of total protein and each protein of interest contained in the DTT fractions after correcting for the 5 × concentration factors for the immediate, 3-min, and 15-min groups. All values represent the means ± SD for *N* = 5. Brains were experimentally paired by processing one brain from each group on the same day. ^a^*P* < 0.01 for paired t-test comparisons of the extents of disulfide bond formation in alpha-enolase compared to gamma-enolase from immediately-frozen brains. ^b^*P* < 0.05 for paired t-test comparisons of the extents of disulfide bond formation in total protein and in alpha-enolase from the 3-min group compared to the immediately-frozen group. ^c^*P* = 0.052 for comparison of the extents of disulfide bond formation in creatine kinase B in the 3 min group to the immediately-frozen group. **D** Glyceraldehyde-3-phosphate dehydrogenase was detected in the DTT fractions but formed aggregates that prohibited estimation of the extents of disulfide bond formation

Figure 1A shows the protein content, by SDS-PAGE, of the pre-column (Pre), flow-through (FT; contains unbound protein), last wash (LW), and DTT-eluted fractions obtained by separation, by PAO-affinity chromatography, of a representative 100,000 g supernatant from the brain of a young healthy SD rat. To aid visualization and measurement of the formerly disulfide-bonded proteins, the DTT-eluted fractions were concentrated fivefold relative to the pre-column fractions, throughout the study, by collecting them in DTT-containing buffer that was one-fifth the volume of the samples loaded onto the columns. The prominent band in the DTT fraction at about 70 kDa was identified by us previously as albumin (Foley et al. 2010), an extracellular protein which contains 17 structural disulfide bonds and effectively serves as a positive internal control for the redox PAO-affinity method. The near absence of protein in the DTT fractions when TCEP was omitted from the immobilized PAO columns demonstrates that unoxidized, PAO-binding, protein thiols had been effectively alkylated by NEM. Determination of the ratio of protein in the DTT-eluted fractions (disulfide bond-containing) to pre-column fractions (total protein), by the Coomassie Blue assay, indicated that 5.4 ± 1.6% (mean ± SD, *N* = 5) of total protein contained reducible disulfide bonds after correcting for the 5 × concentrations of protein in the DTT fractions. This value compares well to those we have reported previously (Foley et al. 2014, 2020), further supporting the notion that reversible oxidations of protein thiols occurs selectively in the healthy brain.

The presence of the enzymes of interest in a representative DTT-eluted fraction was confirmed by LC–MS/MS (Table 1) prior to proceeding with estimations of the extents of disulfide bonding in these enzymes by western blotting. Differential thiol labeling identified cysteine residues in creatine kinase B (CKB) (C141, C146) and in the alpha and gamma subunit isoforms of enolase (C119) as likely PAO-binding thiols that were reversibly oxidized to presumed disulfide bonds. A complete list of proteins identified in the DTT fraction is available in Table S1.

**Table 1:** Enzymes of interest identified in the DTT fraction by mass spectrometry

Protein	Accession Number	Gene	MW (kDa)	SpC	%Cov	Cys(ox)
Creatine kinase B-type	P07335	Ckb	43	42	50	141;146
ATP synthase subunit alpha, mitochondrial	P15999	Atp5f1a	60	33	39	ND
Na^+^/K^+^-transporting ATPase subunit alpha	P06687	Atp1a3	112	22	18	ND
	P06686	Atp1a2	112	17	15	ND
	P06685	Atp1a1	113	16	13	ND
Glyceraldehyde-3-phosphate dehydrogenase	P04797	Gapdh	36	6	20	ND
Gamma-enolase	P07323	Eno2	47	5	12	119
Alpha-enolase	P04764	Eno1	47	5	11	119

DTT-eluted proteins from the fractionation, by redox PAO-affinity chromatography, of a representative brain from the immediately frozen group was run onto a gel but not resolved. The unresolved sample band was excised and shipped to MS Bioworks LLC (Ann Arbor, MI) for alkylation of reduced thiols with iodoacetamide, in-gel tryptic digestion, and protein identification by MS/MS. Data pertaining to only the proteins of interest to this study are summarized. The proteins are ranked by total mass spectral counts (SpC). Reversibly oxidized cysteine residues (Cys_ox_) were identified by differential thiol labeling involving alkylation of (reduced) protein thiols with NEM and alkylation of newly available (formerly oxidized) thiols with iodoacetamide following disulfide bond reduction. Oxidized cysteine residues were defined as those labeled uniquely with iodoacetamide resulting in carbamidomethylation. ND, not detected among the peptides identified and sequenced. %Cov = percent coverage. The complete list of proteins identified by MS/MS in the DTT fraction is provided in Table S1

The extents of disulfide bonding in the specific enzymes of interest were estimated, following western blotting, by determining the ratios of protein band intensities in the DTT-eluted fractions to the intensities in the pre-column fractions and correcting for the 5 × concentrations of the DTT fractions noted above. Relatively large fractions of CKB (19%) and alpha-enolase (22%) contained operationally-defined disulfide bonds (Fig. 1B and C). In contrast, much lower extents of disulfide bonding were determined for the alpha subunit of mitochondrial ATP synthase (9.1%) and gamma-enolase (3.0%). And essentially no alpha subunit(s) of the Na^+^, K^+^-ATPase could be detected in the DTT fractions without over-development of the blots. Intentional delays to freezing of the brains, which models global ischemia (Traystman 2003), generally decreased the extents of disulfide bond content at 3 min but not at 15 min (Fig. 1C). The decreases in disulfide bond content following the 3 -min delays to freezing were statistically significant for total protein (22%) and for alpha-enolase (40%) and bordered on significant for CKB (40%; *P* = 0.052). These findings support the conclusions that alpha-enolase and CKB are highly enriched in the disulfide bond-forming protein fraction from brain and that ischemia can trigger transient reductive shifts in brain protein thiol redox states.

We have previously identified C150 and C154 of GAPDH as PAO-binding vicinal thiols able to undergo oxidation to a disulfide bond (Foley et al. 2020). The presence of GAPDH in the DTT fractions from the immediately frozen brains was confirmed by both mass spectrometry (Table 1) and western blotting (Fig. 1D). However, further consideration of GAPDH vicinal thiols as probes of tissue redox states was prohibited in this study by the complete loss of GAPDH monomer in the DTT fraction and a corresponding appearance of diffuse higher-molecular-weight immunoreactivity in the 70–75 kDa range, consistent with aggregation to form SDS- and DTT-resistant dimers (Fig. 1D). The propensity of GAPDH to undergo aggregation following thiol modifications, including oxidations, is well established (Nakajima et al. 2009; Zaffagnini et al. 2019).

### Protein thiol redox states are stable throughout the lifespan of rats

**Fig. 2:**
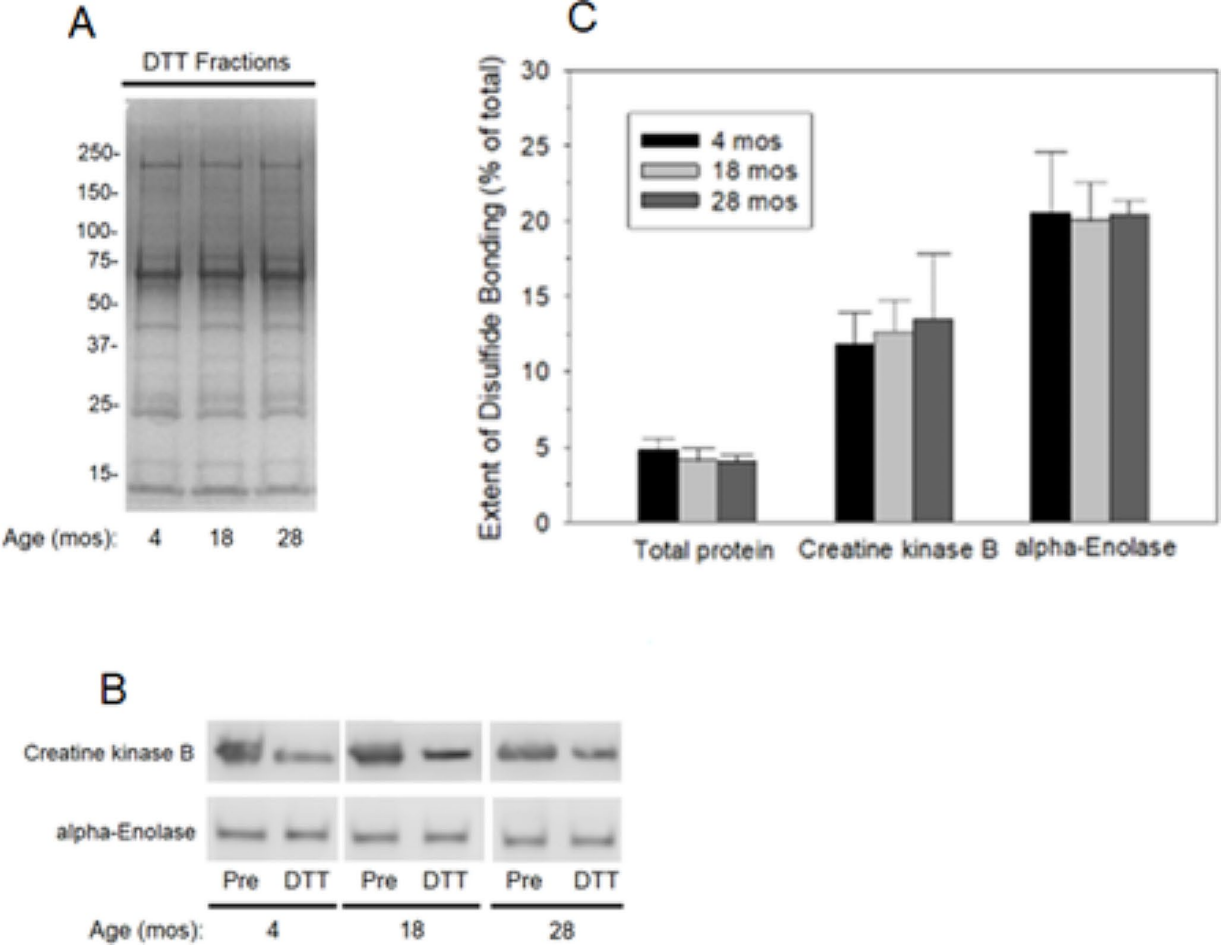
Brain protein thiol redox states are stable through the lifespan of rats. Brains from F344 rats, of 4, 18, and 28 months old, were obtained from the NIA and fractionated by redox PAO-affinity chromatography as described in the legend to Fig. 1. **A** Comparison of the total protein compositions of representative DTT fractions, resolved by protein gel electrophoresis and detected by staining with Imperial™ stain, from brains of the aged groups under study. **B** Western blot comparisons of creatine kinase B and alpha-enolase in representative Pre and DTT fractions. **C** Quantitative comparisons of the extents of disulfide bond formation in total protein, creatine kinase, and alpha-enolase. Data are presented as the means ± SD for *N* = 3–5

Brains from 4-, 18-, and 24-month old F344 rats, representing young adult, middle-aged, and old animals, respectively, were provided by the NIA to support an initial investigation of the effects of animal age on protein thiol redox states in the brain. Fractionation of the proteins from these brains by redox PAO-affinity chromatography showed that protein thiol redox states for total protein (Fig. 2A and C), and for both CKB and alpha-enolase (Fig. 2B and C), were unchanged over the lifespan of these animals.

### Prx-2 can form interprotein disulfide bonds with brain proteins

**Fig. 3:**
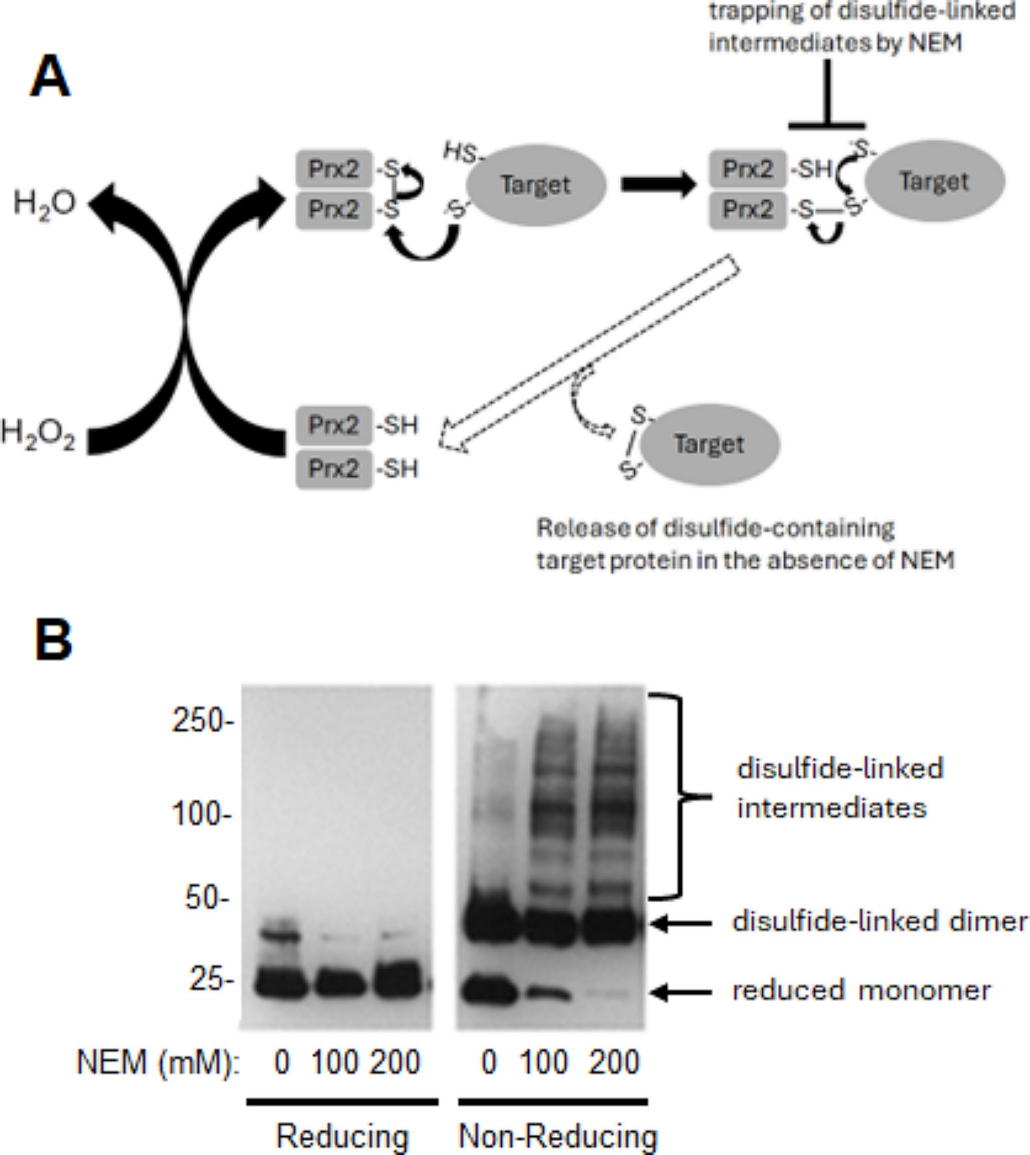
Prx-2 can form disulfide crosslinks with brain proteins. **A** The mechanism by which Prx-2 can transfer, via thiol-disulfide exchange, oxidizing equivalents from hydrogen peroxide to target proteins containing closely-spaced pairs of thiols, resulting in disulfide bond formation in the target proteins. In this pathway, proteins containing dithiols effectively substitute for thioredoxin as reducing co-substrate. **B** A representative Prx-2 redox blot performed twice with similar results. Brains were homogenized in 20 mM acetic acid, pH 4.0, prior to addition of NEM to achieve the indicated final concentrations. Following alkylation and centrifugation at 100,000 × g, western blots for Prx-2 using the resulting supernatant were run in the absence (non-reducing) and presence (reducing) of DTT. Positions of reduced Prx-2 monomers, disulfide (SS)-linked Prx-2 dimers, and high molecular weight disulfide-linked complexes between Prx-2 and other brain proteins, required intermediates in Prx-2-catalyzed protein disulfide bond formation, are indicated

The pathways of protein disulfide bond formation are an important consideration. The two-Cys subtype of Prxs have been implicated, in limited studies, as possible catalysts of protein disulfide bond formation in proteins (Jarvis et al. 2012). The mechanism is purported to involve transfer, by thiol-disulfide exchange, of oxidizing equivalents from hydrogen peroxide to pairs of protein thiols that are closely-spaced as a result of primary, tertiary, or quaternary structures (Fig. 3A). In principle, such dithiol motifs on many proteins, substituting for the catalytic dithiols of thioredoxins as reducing co-substrates, may be oxidized by Prxs. A key requirement for such catalysis is the formation of an intermediate interprotein disulfide bond between the Prx and the protein substrate. Such interprotein disulfides might not be detected, however, as they would be readily “resolved” (i.e., cleaved) by the second thiol on the protein substrate on route to protein disulfide bond formation or, in an unproductive reaction, by the second, so-called resolving thiol of the Prx (Fig. 3A).

Here, we performed redox western blots for Prx-2, an abundant Prx isoform in brain tissue, to determine if disulfide-linked complexes between Prx-2 and other brain proteins could be detected. Like other classical 2-Cys Prxs, the basic functional unit of Prx-2 is a homodimer that can exist in fully reduced and oxidized forms which migrate as a monomer and a disulfide-linked dimer, respectively, under denaturing conditions. These experiments involved treatment of rat brain homogenates with and without NEM which, we predicted, should trap already-formed interprotein disulfide intermediates by alkylating reduced thiols on protein substrates that were proximal to the disulfide bond and/or the resolving thiol of Prx-2 (Fig. 3A).

Figure 3B shows that, when protein samples were incubated with DTT, Prx-2 migrated on the gel/blot, as expected, mainly in the reduced (monomer) form under denaturing conditions, at all concentrations of NEM (Fig. 3A). Also as anticipated, Prx-2 migrated mostly as either the reduced monomer or the oxidized dimer under non-reducing conditions (i.e., the absence of DTT) in samples not treated with NEM. Importantly, however, some higher-molecular-weight (HMW) immunoreactivity for Prx-2, spanning approximately the 50–250 kDa range of the blot, and indicative of interprotein disulfide bonds between Prx-2 and brain proteins of varying size, was evident in this sample. As predicted, the intensity of these HMW forms of Prx-2 increased markedly under non-reducing conditions in samples treated with NEM. Moreover, the increase in the HMW forms of Prx-2 induced by NEM coincided with a large decrease in the reduced (monomer) form of Prx-2, which would be expected to result from alkylation of nearby thiols on Prx-2-targeted proteins, thus preventing the release of reduced Prx-2 (Fig. 3A). Detection of the HMW forms of Prx-2, spanning 50–250 kDa, under non-reducing but not reducing conditions argues strongly that Prx-2 can form interprotein disulfide bonds with apparently many different brain proteins. These findings are consistent with the possibility that Prx-2, and perhaps related two-Cys Prxs, can couple the removal of hydrogen peroxide to the incorporation of potential regulatory disulfide bonds in proteins. Such a pathway would obviate the need for direct, and typically sluggish, reactions of target thiols with hydrogen peroxide while also underpinning the kinetic competence, and potentially the specificity, required for cellular signaling.

## Discussion

Protein dithiol-disulfide transitions underlie catalytic maintenance of redox homeostasis by redoxin enzymes (Holmgren et al. 2005), including Prxs, may be key players in cellular redox buffering (Hansen et al. 2009), and have been implicated by us as plausible regulatory switches on diverse proteins (Foley et al. 2014, 2016, 2020). The present study examined the extents of oxidations of PAO-binding vicinal thiols on selected non-peroxidase enzymes as *reporters* of redox metabolism in brains from (i) young healthy SD rats, (ii) young adult, middle-aged, and old F344 rats, and (iii) young healthy SD rats that were subjected to intentional, short-term, delays between euthanasia, by decapitation, and freezing of the brains to permit metabolism associated with global ischemia (Traystman 2003). In all cases, oxidations of protein thiols during experimental procedures were blocked by alkylation of thiols by NEM. In addition, experiments were performed to investigate the possibility that redox states of vicinal thiols on brain proteins may be coupled to the activity of Prx-2, a 2-Cys subtype of peroxiredoxins abundantly expressed in the brain.

### PAO-binding thiols of alpha-enolase and CKB are preferentially oxidized in healthy brains

The relatively high fractions of alpha-enolase (22%) and CKB (19%) found here to contain thiols that had been reversibly oxidized to disulfide bonds, compared to the other enzymes under study, and the stability of the monomers of these enzymes, in contrast to GAPDH, highlighted these enzymes as potentially useful intrinsic probes of changes in tissue redox states in aging and disease. The significance of oxidations of the PAO-binding vicinal thiols on alpha-enolase and CKB for the activities of these enzymes is outside the scope of the present study and remains to be investigated. Previous studies have linked oxidations of thiols on CKB (Mekhfi et al. 1996; Wolosker et al. 1996; Konorev et al. 1998; Hurne et al. 2000; Reddy et al. 2000; Zhao et al. 2007) and alpha-enolase (Fratelli et al. 2002; Shenton and Grant 2003; Ishii and Uchida 2004) to lower enzyme activities. That C146 was identified as an important site of oxidative inhibition of CKB activity (Zhao et al. 2007) is noteworthy in light of the present findings which identified the closely-spaced thiols at C141 and C146 as PAO-binding vicinal thiols of CKB that were oxidized to presumed disulfide bonds.

### Extents of oxidations of PAO-binding thiols on alpha- and gamma- enolases suggest more oxidizing thiol-linked redox potentials operating in astrocytes than in neurons in the brain

The difference in extents of oxidation of the alpha and gamma subunit isoforms of enolase reported here is striking. Brain expresses the alpha subunit isoform of enolase in both neurons and glia, the most abundant subtype of which are astrocytes (Miller 2018), while the gamma isoform is specific to neurons (Schmechel et al. 1978). Importantly, all six of the cysteine residues of alpha-enolase, including the two identified here as probable sites of oxidation, are also present in the gamma isozyme (UniProt Consortium 2023). We suggest that the much higher oxidation of alpha-enolase (22%) compared to gamma-enolase (3%) may be explained by previously unrecognized more oxidizing protein thiol-linked redox potentials in astrocytes compared to neurons. Indeed, astrocytes produce much more ROS than do neurons (Lopez-Fabuel et al. 2016; Vinokurov et al. 2021). Moreover, reported values from one study for reduced (GSH) and oxidized (GSSG) glutathione allow calculations of GSH/GSSG ratios, evidently overlooked by the authors, that are *86-fold more oxidizing* in astrocytes than neurons (Makar et al. 1994). The present result showing a high extent of oxidation of thiols on CKB, which is also more enriched in astrocytes than in neurons (Molloy et al. 1992), is consistent with this view. In addition to high rates of ROS generation, additional metabolic features of astrocytes that may promote more oxidizing protein thiol redox potentials include (i) the uptake, via the X_C_^−^ antiporter, and NADPH-dependent reduction of cystine to produce cysteine, a precursor of GSH synthesis, and (ii) the export of GSH (Pérez-Sala and Pajares 2023). The coupling of these activities would promote the net export of reducing equivalents. Thus, the present findings call further attention to the cellular compartmentalization of metabolism in the brain (Almeida et al. 2023).

### Brain protein vicinal thiol redox states are stable over the lifespan of rats

Ill-defined oxidative stresses centered on irreversible, free radical-mediated, oxidations of biomolecules, of uncertain functional significance, have generally been assumed to increase in the brain with age and, even more so, with the development of aging-related neurodegenerative disorders (Foley 2019). However, the effects of aging on the redox states of protein thiols in the brain, particularly the dithiol-disulfide redox couples investigated here, have not been well studied. The present findings that extents of disulfide bond formation in total protein, and in both CKB and alpha-enolase, did not differ in brain extracts from 4, 18, and 28 month-old F344 rats argue that protein thiol redox states are strongly buffered over the lifetime of these animals. Thus, any protein thiol-directed oxidative stresses arising during aging must be matched by the protein disulfide bond-reducing activities of the thioredoxin and glutathione systems. These results, although focused on protein dithiol-disulfide redox couples, agree with findings by others that bulk protein thiol redox states are resistant to aging-related perturbations in the brain (Xiao et al. 2020) and skeletal muscle (Tohma et al. 2014) of mice and in Drosophila (Menger et al. 2015). Notably, protein thiol redox states are maintained in the brain during aging despite reported aging-related increases in the expression of the thioredoxin interacting protein (Ismael et al. 2021), an inhibitor of thioredoxin. Lower thioredoxin activity might increase Prx-catalyzed oxidations of vicinal thiols on proteins other than thioredoxins, as suggested by the present findings, and would require a greater flow of electrons from glutathione to support reductions of the resulting protein disulfides (Du et al. 2012). In this light, it is noteworthy that ratios of reduced to oxidized glutathione decrease while protein S-glutathionylation increases in the brain as a function of animal age (Rebrin et al. 2007), consistent with an increased oxidative stress on the glutathione system.

### Decreases in protein disulfides following delayed freezing of brains reveals a transient reductive shift in brain redox metabolism following the onset of ischemia

Short-term delays to the freezing of collected brains, following decapitation, were intentionally performed here as a simple model to test the effects of global ischemia-triggered metabolic perturbations on protein vicinal thiol redox states. The finding that a delay of 3 min, although not 15 min, decreased the ratios of oxidized to reduced protein thiols establishes, by definition, a *reductive shift* in protein thiol redox potentials in the early moments following cessation of blood flow to the brain. A reductive shift may be predicted to occur in ischemic tissues simply owing to the depletion of oxygen and a corresponding decrease in hydrogen peroxide levels, which are suggested by the present findings to be linked to protein vicinal thiol redox states by Prx activity. However, numerous studies report more complex changes in redox metabolism in ischemic-hypoxic tissues and cells, including bursts of ROS production, even in the absence of re-oxygenation (Clanton 2006; Smith et al. 2017). Such bursts may produce temporary compensatory increases in the reducing activities of the thioredoxin and glutathione systems by stimulating NADPH production coupled to oxidation of glucose by the pentose phosphate pathway (Rasler et al. 2007). Importantly, astrocytes store limited supplies of glycogen that can support glucose metabolism in the early moments following ischemia (Cai et al. 2020). The apparent increases in extents of protein thiol oxidations observed here from 3 to 15 min is consistent with a number of findings, including our own (Foley et al. 2014), that oxidative stress can ensue in the postmortem brain following longer delays to freezing (Harish et al. 2011; Heales et al. 2011; Eckman et al. 2018). While the mechanisms by which tissue ischemia can promote oxidative changes in the absence of re-oxygenation remain to be established, the results of the present study demonstrate that brain ischemic metabolism has the capacity to promote, in the short-term, reductive compensation and, potentially, reductive stress. Moreover, they suggest that changes in protein thiol redox states may be underappreciated mediators of outcomes of ischemic insults.

### Peroxiredoxins may link protein vicinal thiol redox states to hydrogen peroxide removal in the brain

The trapping, by NEM, of disulfide-linked complexes formed between Prx-2 and apparently a wide range of brain proteins demonstrated here is in agreement with an ability of the two-Cys subtype of Prxs to catalyze dithiol-disulfide transitions in proteins other than thioredoxin (Jarvis et al. 2012). In this scenario, proteins containing closely-spaced thiol pairs substitute for thioredoxin as reducing co-substate for the removal of hydrogen peroxide. The observation that the trapping, by NEM, of these interprotein disulfides corresponded with a decrease in the reduced (monomeric) form of Prx-2 is consistent with a mechanism involving alkylation of thiols on the target proteins that had access to the disulfide-linked sulfur atoms bridging Prx-2 and the target proteins, thereby preventing attack on the intermediate disulfides by these thiols and the subsequent release of reduced Prx-2. The coupling, by Prxs, of hydrogen peroxide removal to disulfide bond formation in target proteins would obviate the need for the thiols on target proteins to outcompete Prxs for oxidation by hydrogen peroxide and provide kinetically-competent pathways for redox buffering by vicinal thiol motifs on proteins other than thioredoxin and for potential regulatory disulfide bond formation in at least some proteins. Given the unusually high reactivity of Prxs with adventitious hydrogen peroxide (Peskin et al. 2007), however, we cannot exclude the possibility that many of the interprotein disulfides trapped by NEM formed in the homogenate, rather than in the brain, despite homogenization of the brains in these experiments at pH 4 to prevent or slow thiol oxidations. Whether formed in the brain or in the homogenates, these results clearly demonstrate the potential for Prx-2, and possibly other two-Cys Prxs, to catalyze the incorporation of disulfide bonds in brain proteins by thiol-disulfide exchange.

In summary, measures of the extents of oxidations, to presumed disulfide bonds, of PAO-binding vicinal thiols on non-peroxidase proteins support novel and unexpected characteristics of brain redox metabolism in health, aging, and disease. Specifically, marked difference in the extents of oxidations of thiols on the alpha- and gamma- subunit isoforms of enolase support underappreciated differences in protein thiol-linked redox states of astrocytes and neurons, in vivo. Moreover, contrary to assumptions that the brain is subject to global oxidative stress during aging, brain protein thiol redox states remained stable throughout the lifespan of rats. The resistance of protein thiol redox states to change during aging combined with the reductive shift in these redox states in the early moments following global ischemia highlight a robust capacity of brain redox metabolism to avert bulk oxidations of protein thiols. Finally, the trapping of disulfide-linked complexes between Prx-2 and brain proteins suggests pathways by which protein vicinal thiol redox states may be coupled to redoxin-mediated redox homeostasis.

### Supplementary Information

Below is the link to the electronic supplementary material.

Supplementary file1 (XLSX 24 kb)

Supplementary file2 (DOCX 164 kb)

## Data Availability

No datasets were generated or analysed during the current study.

## Abbreviations

CKB: Creatine kinase B
DTT: Dithiothreitol
F344: Fischer 344
FT: Flow-through
GAPDH: Glyceraldehyde-3-phosphate dehydrogenase
LW: Last wash
NEM: N-ethylmaleimide
NIA: National Institute on Aging
PAO: Phenylarsine oxide
Prx: Peroxiredoxin
Redox: Oxido-reductive
ROS: Reactive oxygen species
SD: Sprague-Dawley
TCEP: Tris(2-carboxyethyl)phosphine
