# Supplementary material for: Protein vicinal thiols as intrinsic probes of brain redox states in health, aging, and ischemia
Source: Metab Brain Dis. 2024 Jun 7;39(5):929–40. doi: 10.1007/s11011-024-01370-3 (PMC11233328; doi:10.1007/s11011-024-01370-3)
Supplement: Supplementary file 2 — Supplementary file2 (DOCX 164 kb) [file 11011_2024_1370_MOESM2_ESM.docx]

**Supplemental Figures S1-S5**

**
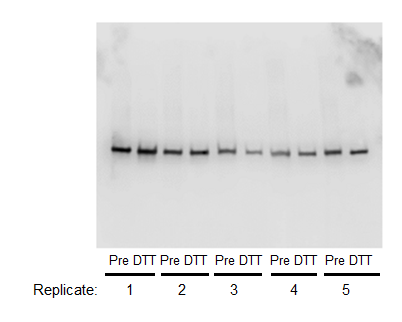
**

**Fig. S1. Extents of disulfide bond formation involving creatine kinase B following immediate freezing of brain tissue.** 100,000 x g supernatants from the brains of Sprague Dawley rats were fractionated by redox PAO-affinity chromatography to obtain the DTT-eluted, disulfide bond-forming, protein fraction as described in the Materials and Methods. The extents of disulfide bonding involving creatine kinase B (42.7 kDa) was estimated by western blot comparisons of the intensities of the bands in the DTT (disulfide bond-forming) and Pre-column (total protein) fractions. All five biological replicates are shown.

**
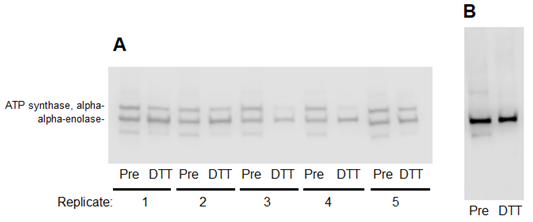
**

**Fig. S2. Extents of disulfide bond formation involving the alpha subunit of mitochondrial ATP synthase and alpha-enolase following immediate freezing of brain tissue.** 100,000 x g supernatants from the brains of Sprague Dawley rats were fractionated by redox PAO-affinity chromatography to obtain the DTT-eluted, disulfide bond-forming, protein fraction as described in the Materials and Methods. A) The extents of disulfide bonding involving the alpha subunit of mitochondrial ATP synthase (59.8 kDa) and alpha-enolase (47.1 kDa) were estimated by western blot comparisons of the intensities of the bands for each of these proteins in the DTT (disulfide bond-forming) and Pre-column (total protein) fractions. All five biological replicates are shown. B) A western blot of the Pre and DTT fractions from a representative brain (replicate 5) probed only for alpha-enolase to show the absence of the band for the alpha subunit of the ATP synthase.


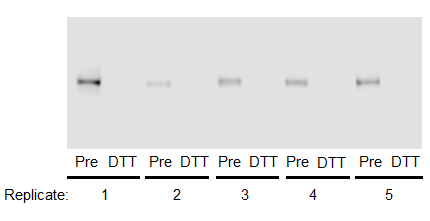


**Fig. S3. Extents of disulfide bond formation involving the alpha subunit of Na^+^/K^+^-ATPase following immediate freezing of brain tissue.** 100,000 x g supernatants from the brains of Sprague Dawley rats were fractionated by redox PAO-affinity chromatography to obtain the DTT-eluted, disulfide bond-forming, protein fraction as described in the Materials and Methods. The extents of disulfide bonding involving the alpha subunit of Na^+^/K^+^-ATPase (112-113 kDa) was estimated by western blot comparisons of the intensities of the bands in the DTT (disulfide bond-forming) and Pre-column (total protein) fractions. All five biological replicates are shown.


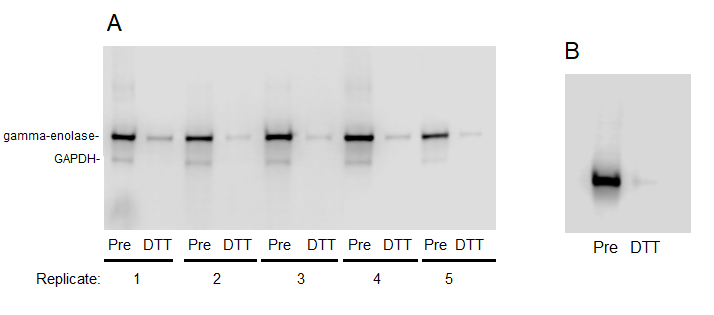


**Fig. S4. Extents of disulfide bond formation involving gamma-enolase and glyceraldehyde-3-phosphate dehydrogenase (GAPDH) following immediate freezing of brain tissue.** 100,000 x g supernatants from the brains of Sprague Dawley rats were fractionated by redox PAO-affinity chromatography to obtain the DTT-eluted, disulfide bond-forming, protein fraction as described in the Materials and Methods. A) The extents of disulfide bonding involving gamma-enolase (47.1 kDa) and GAPDH (35.8 kDa) were estimated by western blot comparisons of the intensities of the bands for each of these proteins in the DTT (disulfide bond-forming) and Pre-column (total protein) fractions. All five biological replicates are shown. B) A western blot of the Pre and DTT fractions from a representative brain (replicate 5) probed only for gamma-enolase to show the absence of the band for GAPDH. As reported in the Results, stronger development of GAPDH blots revealed aggregation, which precluded estimation of the extents of disulfide bond formation involving this protein.

**
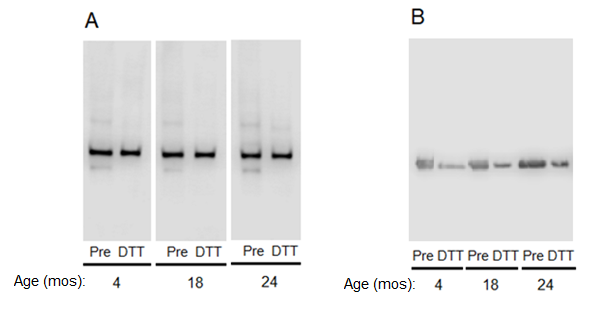
**

**Fig. S5. Extents of disulfide bond formation involving A) alpha-enolase and B) creatine kinase B from the brain as a function of animal age.** 100,000 x g supernatants from the brains of 4, 18, and 24 mos-old F344 rats were fractionated by redox PAO-affinity chromatography to obtain the DTT-eluted, disulfide bond-forming, protein fractions as described in the Materials and Methods. The extents of disulfide bonding involving A) alpha-enolase (47.1 kDa) and creatine kinase B (42.7 kDa) were estimated by western blot comparisons of the intensities of the bands for each of these proteins in the DTT (disulfide bond-forming) and Pre-column (total protein) fractions. Representative fractions from each of the three age groups are shown. Empty lanes in between in the gel probed for in blot A were removed, indicated by the spacing.
